# Microscopic Examination of Polymeric Monoguanidine, Hydrochloride-Induced Cell Membrane Damage in Multidrug-Resistant *Pseudomonas aeruginosa*

**DOI:** 10.3390/polym9090398

**Published:** 2017-08-31

**Authors:** Xun Cao, Lu Meng, Niya Zhang, Zhongxin Zhou

**Affiliations:** 1 College of Animal Sciences & Technology, Huazhong Agriculture University, 1 Shizishan Street, Wuhan 430070, China; caxu1228@sina.com (X.C.); mengl1003@sina.com (L.M.); zhangniya@mail.hzau.edu.cn (N.Z.); 2 The Cooperative Innovation Center for Sustainable Pig Production, 1 Shizishan Street, Wuhan 430070, China

**Keywords:** polymeric monoguanidine, antibiotics resistant, *Pseudomonas aeruginosa*, membrane

## Abstract

Advances in antimicrobial activities of molecule-containing, multiple guanidinium groups against antibiotics-resistant bacteria should be noted. The synthesized polyoctamethylene monoguanidine hydrochloride (POGH), carrying cationic amphiphilic moieties, display excellent activity against multidrug-resistant *Pseudomonas aeruginosa* (MDR-PA) and other antibiotics-resistant bacteria. The membrane damage effects of POGH on MDR-PA were clarified using beta-lactamase activity assay, confocal fluorescence microscopy, scanning electron microscopy, and transmission electron microscopy. The results showed that POGH disrupted both the outer and inner membranes and the intracellular structure of MDR-PA to different extents depending on the dose. All concentrations of POGH within 3–23 μg/mL increased the outer membrane permeability, which facilitated the release of beta-lactamase across the inner membrane. A median dose (10 μg/mL) of POGH led to the separation of the inner and outer membrane, an increase in the membrane gap, and outer membrane structure damage with still maintained overall cytoskeletal structures. The application of a 30 μg/mL dose of POGH led to the collapse of the outer membrane, cellular wrinkling, and shrinkage, and the formation of local membrane holes. The disruption of the outer and inner membranes and the formation of the local membrane holes by a relative high dose were probably the main bactericidal mechanism of POGH. The microscopic evidence explained the strong outer-membrane permeation ability of guanidine-based antimicrobial polymers, which could be considered for the molecular design of novel guanidine-based polymers, as well as the damaged membrane structure and intracellular structure of MDR-PA.

## 1. Introduction

New antimicrobials are urgently required to combat the growing health threat posed by antibiotics-resistant bacteria [1,2,3,4]. The bacterial membrane is recently considered as a promising antimicrobial target against Gram-negative bacteria [5,6,7,8], e.g., multidrug resistant *Pseudomonas aeruginosa* (MDR-PA) [5,6,7]. Modifications to lipid membranes of Gram-negative bacteria, e.g., *Pseudomonas aeruginosa* [9], and Gram-positive bacteria, e.g., *Staphylococcus aureus* [10], have partially contributed to their resistance generation compared to the wide type strain.

Guanidine-based antimicrobial polymers [11,12] targeting bacterial membranes show excellent anti-bacterial [13,14,15,16], anti-fungal [17], and anti-viral [18,19] activities. Importantly, recent encouraging advances in combating antibiotics-resistant bacteria [20,21,22,23] have received considerable attention. Antimicrobial, molecule-containing, multiple guanidinium groups were endowed with activities against antibiotics resistant bacteria. For example, guanidinylation, of the polycationic headgroup in neomycin B, enhances the in vitro antibacterial activity against a neomycin B-, kanamycin A-, and gentamicin-resistant *Pseudomonas aeruginosa* [21]. Guanidinylation, of the polycationic headgroup in kanamycin A, with weak activity against methicillin-resistant *Staphylococcus aureus* (MRSA), restores the in vitro anti-MRSA activity [21]. The synthesized paraguanidinoethylcalix[4]arene and chlorhexidine digluconate, commonly used disinfectants in hospitals, which both contain multiple guanidinium groups, also have good in vitro activity against MRSA and meticillin-resistant, coagulase-negative staphylococci, vancomycin resistant *Enterococcus faecium*, and *Enterobacteriaceae* spp. with resistant phenotypes [22]. 

Polyhexamethylene monoguanidine hydrochloride (PHGH), a well-known cationic amphiphilic polymer, is especially efficient against methicillin resistant-*Staphylococcus aureus* and coagulase-negative staphylococci [20,23] and is recommended as a disinfectant to fight nosocomial infections against methicillin-resistant *Staphylococcus aureus*, *Pseudomonas aeruginosa* [24,25]. The newly synthesized polyoctamethylene monoguanidine hydrochloride (POGH) has significantly lower minimal inhibition concentration values (MIC, 0.5–16 mg/L) against 370 clinical strains, especially 96 isolates of which were antibiotics-resistant, compared to PHGH (MIC, 1–64 mg/L) and chlorhexidine digluconate (MIC, 2–64 mg/L) [23]. Interestingly, POGH displays excellent activity against multidrug resistant *Pseudomonas aeruginosa* (MIC, 8–16 mg/L), meticillin resistant-*Staphylococcus aureus* (MIC, 1–8 mg/L), coagulase-negative staphylococci (MIC, 1–2 mg/L), vancomycin resistant *Enterococcus faecium* (MIC, 2–4 mg/L), ceftazidime resistant-Citrobacter spp. (MIC, 1–4 mg/L) and Enterobacter spp. (MIC, 2–4 mg/L) [23].

Although polymerized guanidine antibacterial agents display great application potential against antibiotics-resistant bacteria, details of their mechanism of action are limited. Studies on the mechanism of action of guanidine antibacterial agents have been primarily focused on polyhexamethylene biguanide (vantocil) [26] and biguanide (alexidine and chlorhexidine) [27] disinfectants. Polyhexamethylene biguanide was reported to disrupt the extracellular matrix and cytoplasmic membrane of *E. coli* ATCC 8739 [26]. Using the phospholipid vesicles as the model cytoplasmic membrane of bacteria, alexidine was suggested to increase cell membrane permeability, which in turn led to the phase separation of phospholipids and the formation of a phospholipid domain on the cell membrane [27]. However, so far, no microscopy studies demonstrating the function of polymerized monoguanidine molecules on the cell membrane of drug-resistant bacteria have been conducted. 

Therefore, to understand the function and mechanism of action of polymerized monoguanidine antibacterial agents against drug resistant bacteria, we used POGH as a representative of polymerized monoguanidine antibacterial agents and studied their disruptive effect on the cell membrane of MDR-PA. Utilizing beta-lactam enzyme activity detection, confocal fluorescence microscopy, field emission scanning electron microscopy, and transmission electron microscopy, we observed the effect of polyoctamethylene guanidine hydrochloride on the organization of bacterial cell membranes.

## 2. Materials and Methods

### 2.1. Polymerized Monoguanidine Antibacterial Agents and Other Chemicals

**Figure 1:**
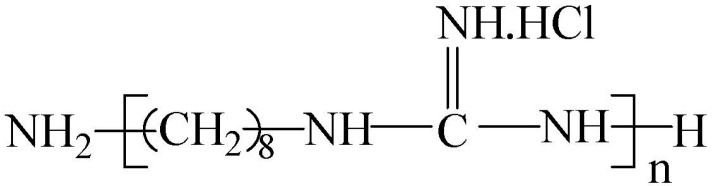
The chemical structure of polyoctamethylene monoguanidine hydrochloride (POGH).

The polyoctamethylene monoguanidine hydrochloride (POGH) was kindly provided by Dr. Dafa Wei in East China University of Science and Technology (Shanghai, China). The chemical structure of POGH is shown in Figure 1. POGH was synthesized based the method suggested by Zhou et al. [23] The number-average molecular weight, weight-average molecular weight, and polydispersity coefficient of POGH are 457, 512, and 1.12, respectively. 

Imipenem and fluorescein isothiocyanate (FITC) were bought from Sigma-Aldrich Co. (Shanghai, China). Other reagents were analytical purity. Deionized water was used in all experiments.

### 2.2. Strains

The multidrug resistant *Pseudomonas aeruginosa* clinical strains (clinical strain 09-696) were provided by Huashan Hospital, ShanghaiMedical College, Fudan University (Shanghai, China). The strains were identified using the Vitek automated identification system (BioMérieux, Marcy l’Etoile, France) and were confirmed by the API-GN system (BioMérieux, Marcy l’Etoile, France). 

### 2.3. Evaluation of Bactericidal Dynamics

Cryopreserved MDR-PA cells (clinical strain 09-696) were thawed out at room temperature and a 20 μL aliquot was added to a 250 mL conical flask containing 40 mL of Luria-Bertani (LB) broth. The flask was incubated on a shaker overnight (200 rpm at 37 °C). The next morning, 200 μL of the bacterial medium was added to a 250 mL conical flask containing 40 mL of fresh LB medium. The bacteria were continuously cultured to an optical density at a wavelength of 600 nm (OD_600_) of 1. The bacteria culture was centrifuged, and the pellet was resuspended in 10 mM PBS (pH 7.4). The centrifugation-resuspension steps were repeated thrice. Based on the data that the density of a bacterial suspension at OD_600_ = 0.35 was 2.5 × 10^8^ colony forming units (CFU)/mL, a bacterial suspension with a density of 6.25 × 10^6^ CFU/mL was prepared. The bacterial suspension was incubated with 32 μg/L of the antibacterial agent at 30 °C. After various time points, 20 μL aliquots of the incubated bacterial suspension were collected, diluted, and plated, and the number of live bacteria after 24 h at 37 °C was counted. Bacterial CFU/mL was correlated with OD_600_ and after 10-fold dilution, in which the concentration of the bacterial suspension was 2.5 × 10^8^ CFU/mL when OD_600_ = 0.35.

### 2.4. Assessment of Outer Membrane Permeability by Beta-Lactamase Enzyme

The outer membrane permeability was measured by quantifying the activity of the released beta-lactamase enzyme, located in the periplasm of Gram-negative bacteria [28,29,30], after permeabilization of the outer membrane of MDR-PA cell. Cryopreserved MDR-PA strain was thawed at room temperature, and a 20-μL aliquot was added to a 250 mL conical flask containing 40 mL of LB medium. The flask was incubated on a shaker overnight (200 rpm at 37 °C). The next day, 200 μL of the overnight cultured bacterial suspension was added into a 250 mL conical flask containing 40 mL of fresh LB medium. After another hour of incubation, 400 μL of 0.1 M Imipenem was added into the flask to induce the expression of beta-lactamase, with a final concentration of 0.2 μg/mL. The bacteria were continuously cultured until OD_600_ = 1. The bacterial suspension was centrifuged, and the pellet was resuspended in 10 mM PBS (pH 7.4). The centrifugation-resuspension step was repeated thrice. Based on the data that the density of the bacterial suspension was 2.5 × 10^8^ CFU/mL, a bacterial suspension with a density of 9 × 10^8^ CFU/mL was prepared. Different concentrations (final concentration, 3, 10, 17, 23 μg/mL) of POGH were added to the bacteria suspensions for 15 min and 60 min at 30 °C. The control group was not treated with POGH, while all the other processes were similar to that of the treatment group. At the indicated time points, the bacterial suspension was centrifuged at 12,000 rpm (9658 g) for 5 min. The supernatant was collected as a crude enzyme solution. The beta-lactamase-containing supernatant was added to nitrocefin (final concentration, 100 μM) at 30 °C in a final volume of 2 mL of assay buffer (50 mM sodium phosphate buffer with pH 7.4) and nitrocefin hydrolysis was measured spectrophotometrically at a wavelength of 482 nm using an ultraviolet/visible spectrophotometer (Unicotm, UV-2100; UNICO Instruments Co., Ltd., Shanghai, China) as reported [28].

### 2.5. Assessment of Membrane Permeability by Confocal Fluorescence Microscopy

Fluorescein isothiocyanate (FITC) was used as the fluorescent dye to examine POGH-induced changes in the membrane permeability of the MDR-PA cell, as reported [31]. FITC is a low-molecular weight (389.4 Da), green, fluorescent dye that does not penetrate into cells with intact cell membranes.

A FITC stock solution (10 mg/mL) was prepared in acetone, and a MDR-PA suspension was prepared as indicated in the “time-bactericidal curve”. A bacterial suspension with a density of 9 × 10^8^ CFU/mL was incubated with 30 μg/mL POGH for 60 min at 30 °C, followed by cycles of centrifugation and resuspension. The bacterial pellet was then resuspended in PBS (pH 7.4), and 20 μL of the suspension was plated on a poly-lysine coated slide. The slide was placed in a tissue culture dish and incubated at 30 °C for 45 min to allow the cells to attach to the slide. Then, 20 mL of PBS-diluted FITC was added to the dish and incubated at 30 °C (FITC final concentration: 6 μg/mL). After 60 min, the slide was washed in 20 mL of 10 mM PBS for 15 min to remove excess FITC, and this washing was repeated thrice. The slide was then washed with deionized water thrice for 1 min each time, and then dried in a 30 °C incubator. The slide was then examined under a reverse fluorescence microscope (Nikon Eclipse E-600; Nikon Corporation, Tokyo, Japan) and a confocal fluorescence microscope (FV-300, IX71; Olympus Optical Co., Ltd., Tokyo, Japan). The confocal fluorescence microscope was equipped with a 405 nm semiconductor laser as an excitation source, and the laser beam was focused on a 1 μm diameter spot using a 60× object lens. The energy density of the focused excitation was 250 W/cm^2^.

### 2.6. Field Emission Scanning Electronic Microscopy

MDR-PA cultures were prepared as described in the bactericidal dynamics experiment. The collected bacteria were resuspended in PBS and adjusted to an OD_600_ = 2.0. Agents were added to the sample, the concentrations of POGH were 10 μg/mL and 30 μg/mL, respectively, and MDR-PA density was 9 × 10^8^ CFU/mL. After 60 min of incubation, the MDR-PA cultures were centrifuged (3000 rpm, 5 min) and then washed with 10 mM PBS (pH 7.4) thrice. The MDR-PA cells were then resuspended in the same volume of PBS, and 20 μL of the suspension was plated on a poly-lysine-coated slide. The slide was incubated at 30 °C for 90 min to allow the cells to attach to the slide. The slide-immobilized cells were fixed with 2.5% (*w*/*v*) glutaraldehyde in 0.1 M PBS, extensively washed with the same buffer, and then dehydrated across a graded ethanol series. After critical point drying and gold coating, the samples were observed on an FEI SIRION 200 field emission SEM (FEI Co., Hillsboro, OR, USA).

### 2.7. Transmission Electron Microscopy

The MDR-PA cultures were prepared as described in the bactericidal dynamics experiment. The collected bacteria were resuspended in PBS at an OD_600_ = 2.0. After 3-fold dilution with POGH solution, the final MDR-PA density was 9 × 10^8^ CFU/mL, and the concentrations of POGH were 10 μg/mL and 30 μg/mL, respectively. After 60 min of incubation, the MDR-PA culture was centrifuged (3000 rpm, 5 min) and then washed with 10 mM PBS (pH 7.4) thrice. The treated cell suspensions were centrifuged, the pellets were fixed with 2.5% (*w*/*v*) glutaraldehyde in 0.1 M PBS and postfixed with 1% OsO_4_. Then, the samples were dehydrated, embedded, and subjected to ultrathin sectioning, followed by uranium and aluminum staining. The control bacteria without POGH was run in the presence of PBS. The prepared samples were observed with a JEM-2100 TEM (Japan Electron Optics Laboratory Co., Ltd, Tokyo, Japan).

## 3. Results

### 3.1. Polyoctamethylene Monoguanidine Hydrochloride (POGH) Has Bactericidal Activity against MDR-PA

**Figure 2:**
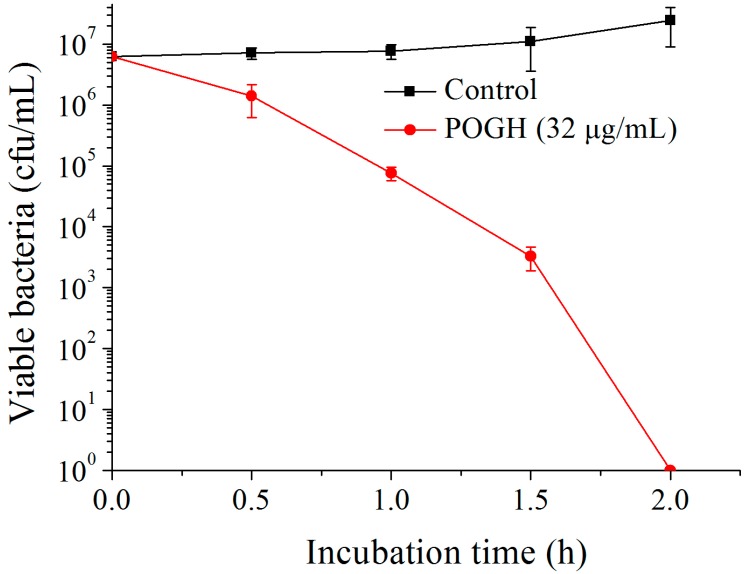
The time-kill curve of polyoctamethylene monoguanidine hydrochloride (POGH) against multidrug-resistant *P. aeruginosa*. Approximately 6.25 × 10^6^ CFU/mL bacterial cells were incubated with 32 μg/mL POGH at 25 °C for 0.5, 1, 1.5, and 2 h. POGH had a minimum inhibitory concentration of 16 μg/mL.

Figure 2 shows that in the bactericidal dynamic experiments, when 6.25 × 10^6^ CFU/mL of MDR-PA was incubated with 32 μg/mL POGH for 2 h, the density of MDR-PA decreased over 6 log_10_ CFU/mL which was indicative of the strong bactericidal activity of POGH against MDR-PA. Previous studies have shown that POGH has an MIC90 value of 16 μg/mL on MDR-PA and its MIC value ranges from 8 to 16 μg/mL [23]. It was reported that AKACID Plus^®^, which is a commercial member of the polymeric guanidine family of disinfectants, had a MIC of 2 μg/mL against *E. coli* and 4 μg/mL of AKACID Plus^®^ killed 10^6^ CFU/mL *E. coli* cells with 1.25 h.

### 3.2. POGH Alters the Permeability of the Outer Membrane of MDR-PA Cell and Induces Beta-Lactamase Release in Periplasm

**Figure 3:**
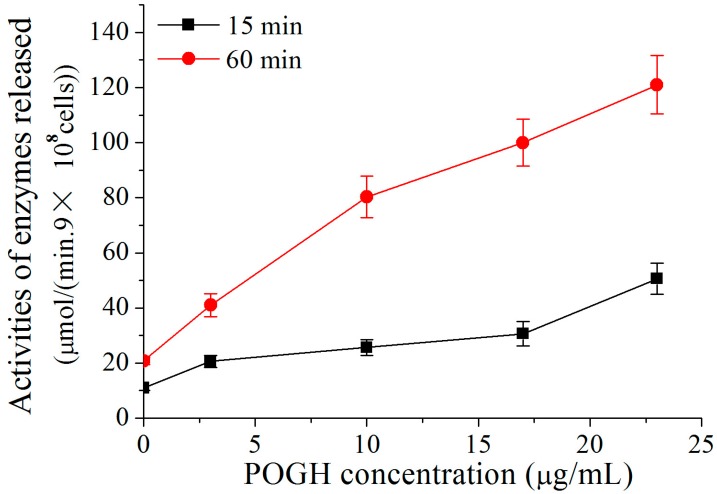
POGH-induced changes in β-lactamase activity. Approximately 9 × 10^8^ CFU/mL multidrug-resistant *P. aeruginosa* cells were incubated with POGH at 30 °C for 15 and 60 min.

To detect damages to the outer membrane permeability of the MDR-PA cell, the activities of the beta-lactamase released across the periplasm of the *P. aeruginosa* cell were measured [28,29]. Because POGH could denature enzymes, POGH was incubated with a concentration of 9 × 10^8^ CFU/mL cells in order to detect enzyme activity. Figure 3 shows that using the same incubation time, higher concentrations of POGH induced higher beta-lactamase activity upon cellular release. When incubated with the same concentration of POGH, longer incubation times led to the secretion of higher amounts of beta-lactamase. This finding indicates that POGH interacts with MDR-PA cells and increases the outer membrane permeability, which in turn leads to the release of beta-lactamase in the periplasm to the extracellular matrix. Moreover, the outer membrane permeability of POGH was strongly correlated with the concentration of antibacterial agents, the incubation time, and the cell concentration.

It is noteworthy that in addition to the observation that a relatively high concentration of POGH increased beta-lactamase activity, all concentrations of POGH within a range of 3–23 μg/mL increased permeabilization of the outer membrane of MDR-PA cell, thereby leading to the release of beta-lactamase to the extracellular matrix. These findings indicated that even a relatively low concentration of POGH could increase the outer membrane permeability. Similar findings were observed using antibacterial peptide analog polymers (concentration range: 0 to 25 μg/mL), and the release of beta-galactosidase to the extracellular matrix and related changes in enzyme activity [32].

### 3.3. Confocal Fluorescence Microscopy Detects an Increase in MDR-PA Membrane Permeability

**Figure 4:**
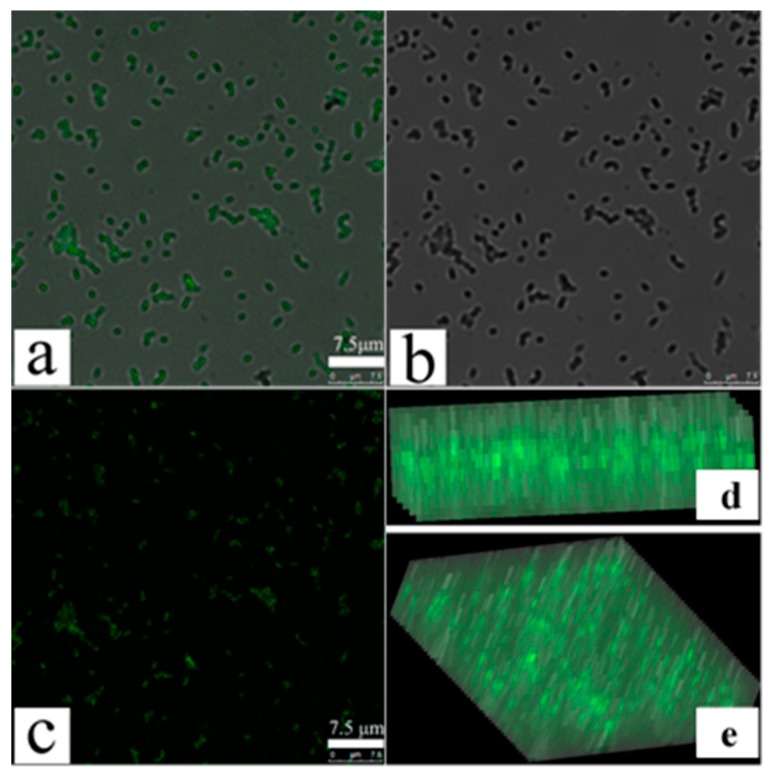
Laser confocal scanning microscopy of fluorescein isothiocyanate (FITC) cellular uptake after permeabilization of bacterial membranes. Multidrug-resistant *P. aeruginosa* cells (9 × 10^8^ CFU/mL) were incubated with 30 μg/mL POGH for 60 min. (**a**) The original image of POGH-treated cells observed by the ordinary fluorescence mode; (**b**) the image observed by the mode of Differential Interference Contrast (DIC); (**c**) the merged image of the corresponding original image and the DIC image; (**d**,**e**) the corresponding images in the *y*–*z* plane.

To further confirm changes in MDR-PA cellular membrane permeability, we performed confocal fluorescence microscopy using FITC fluorescent dye, as reported [31]. FITC does not enter bacterial cells with intact cell membranes, whereas permeabilization of bacterial membranes allows its entry [31]. Figure 4a is the original image of POGH-treated cells observed by the ordinary fluorescence mode. Figure 4b is the image observed by the mode of Differential Interference Contrast (DIC), which means the location of the bacterial cell. Figure 4a coincide completely with Figure 4b to produce the merged Figure 4c image of the corresponding original image and the DIC image, which means the seen fluorescence in Figure 4a come from the bacterial cell. Both Figure 4d,e are the fluorescence observed in the *y*–*z* plane, which shows FITC fluorescence in the intracellular space of the bacterial cell, thereby indicating the membrane permeability of the bacterial cell. Confocal fluorescence microscopy allowed us to determine changes in the membrane permeability. Due to the detection limit of the microscope, aggregation of the FITC in cell was only observed using the relatively high concentration of POGH or the density of bacteria. This differed from the result that a wide concentration rang of POGH could induce the outer membrane permeabilization tested by the release of beta-lactamase in the periplasm to the extracellular matrix.

### 3.4. Scanning Electron Microscopy Shows Changes in MDR-PA Cellular Surface Morphology and Cell Membrane Damage

**Figure 5:**
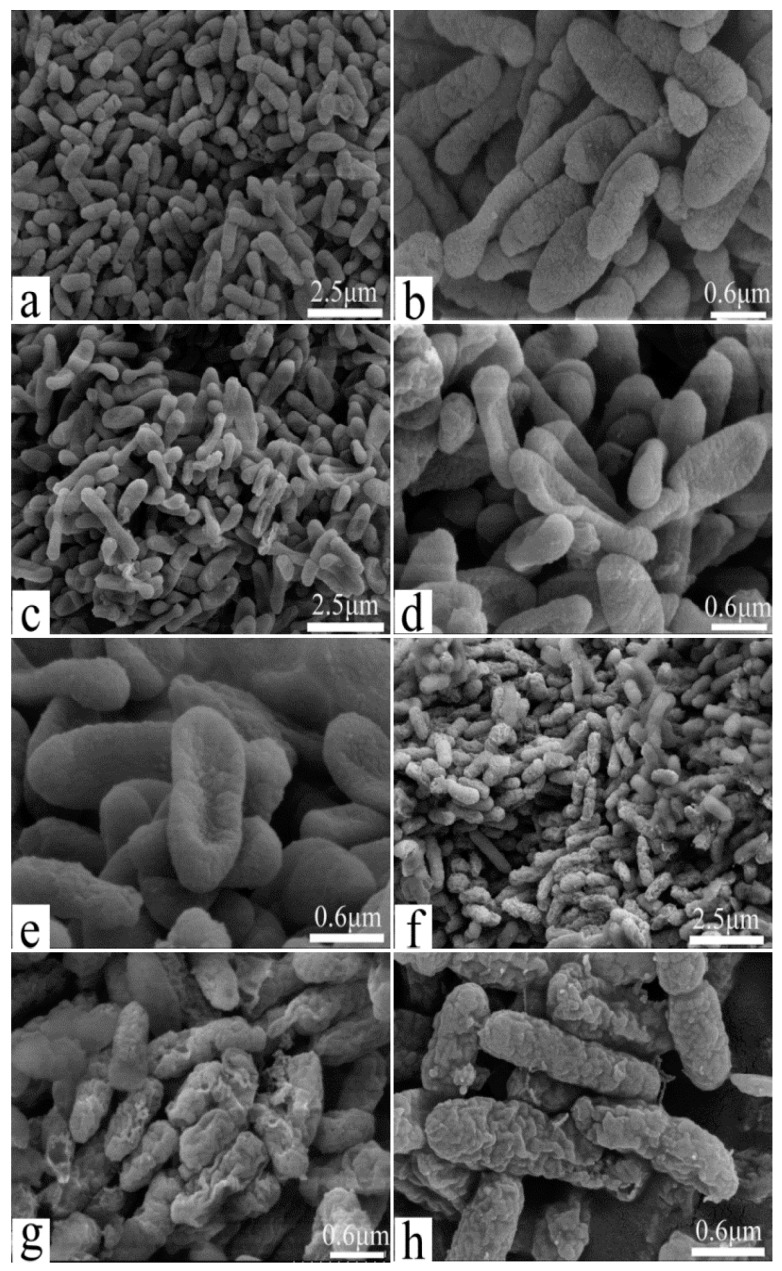
Changes in cell morphology and membrane damage as detected by field emission scanning electron microscopy. Control bacteria (**a**,**b**); approximately 9 × 10^8^ CFU/mL multidrug-resistant *P. aeruginosa* cells were treated with 10 μg/mL (**c**–**e**) POGH for 60 min, or with 30 μg/mL (**f**–**h**) POGH for 60 min. Magnification times ((**a**,**c**,**f**), 4000×; (**b**,**d**,**e**,**g**,**h**), 16,666×).

Field emission scanning electron microscopy was used to directly examine changes in cell surface morphology and cell membrane damage, as reported [33]. Figure 5 shows untreated MDR-PA cells depicting the typical plump and cone-shaped morphology with rough surfaces and complete extracellular coating (Figure 5a,b). After incubation with POGH, the morphology of the MDR-PA cells changed. Using a low POGH concentration (10 μg/mL), the cells were flat in shape (Figure 5c–e), but maintaining the same cytoskeletal structures as that of the untreated control cells. Using a 30 μg/mL dose of POGH, the cells were in a spasm shape, their extracellular surface showed wrinkles, some extracellular coating was missing, and there was cellular debris surrounded the cells (Figure 5f–h), indicating cell membrane damage. No debris was observed in cells exposed to a 10 μg/mL concentration of POGH.

### 3.5. Transmission Electron Microscopy Shows Changes in MDR-PA Intracellular Structure and Cell Membrane Damage

**Figure 6:**
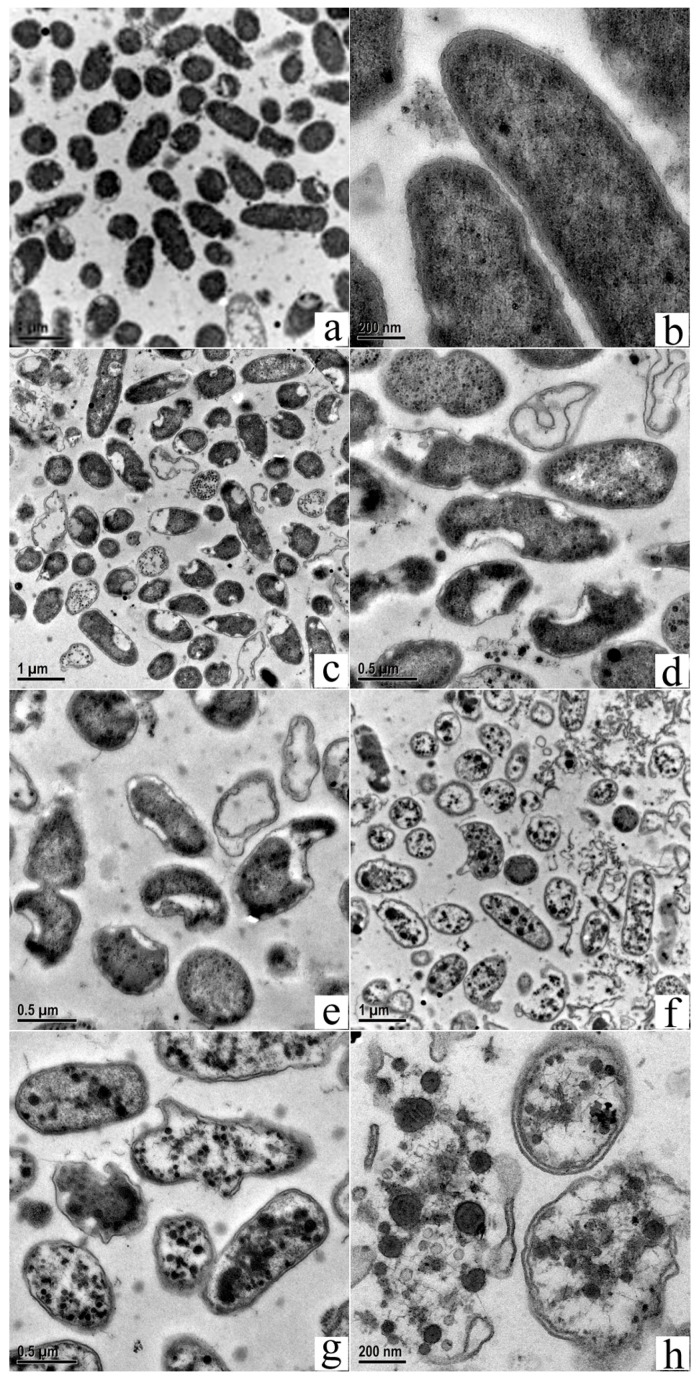
Transmission electron micrographs of multidrug-resistant *P. aeruginosa* (MDR-PA) cells showing membrane damage and changes in the intracellular structure. Control bacteria (**a**,**b**); approximately 9 × 10^8^ CFU/mL MDR-PA cells were treated with 10 μg/mL (**c**–**e**) POGH for 60 min, or with 30 μg/mL (**f**–**h**) POGH for 60 min. Magnification times ((**a**,**c**,**f**), 10,000×; (**d**,**e**,**g**), 20,000×; (**b**,**h**), 50,000×).

Transmission electron microscopy was used to assess changes in intercellular structures, as reported [34]. Figure 6 shows untreated MDR-PA cells with uniform electron density and a clear extracellular outer membrane and intracellular protoplast (Figure 6a,b). On the other hand, MDR-PA, which was exposed to POGH, exhibited damaged membrane and altered intracellular structures. Figure 6c–e shows that cells exposed to 10 μg/mL POGH exhibited a flat-shaped cellular morphology. Their cellular inclusions were apparently shrunken, and the inner and outer membranes depicted segregation, thereby making membrane boundaries clearly visible. Minor aggregated dense particles were observed in the inner membrane protoplast. When cells were exposed to 30 μg/mL POGH (Figure 6f–h), dense condensed particles and large aggregates were observed in the protoplast. Some cells showed extensive wrinkling and concave notches on their outer membrane. The outer membrane structure was disintegrated and collapsed, and the cells were surrounded by a significantly higher amount of cellular debris.

## 4. Discussion

It is interesting that all concentrations of POGH within a range of 3–23 μg/mL increased permeabilization of the outer membrane of the MDR-PA cell, which indicated the strong bactericidal potential and outer-membrane permeabilization ability of the guanidinium, group-based antimicrobial polymer. But it is noteworthy that both the positively charged, guanidinium moieties and the hydrophobic side chain of POGH contribute to its outer-membrane permeabilization ability. 

Scanning and transmission electron microscopy analyses show that the application of a 30 μg/mL concentration of POGH led to the development of concave surfaces on most of the bacteria (Figure 5g,h and Figure 6h). However, compared to the scanning electron micrograms, the concave notches in the transmission electron micrograms were not indicative of membrane damage due to POGH and instead might have been caused by specific steps in specimen preparation. Our scanning electron microscopy and transmission electron microscopy findings indicate that POGH-induced, dose-dependent membrane structure damages were a local effect, and the collapse of membrane structures was not a global effect. The concave collapsed surface observed during scanning electron microscopy analysis and the notch detected during transmission electron microscopy evaluation are probably “membrane holes” that were formed after POGH had caused membrane damage.

Biguanide disinfectant-bis guanidine (alexidine) and chlorhexidine attach to the cell membrane and induce the separation of membrane components, thereby resulting in the segregation of phospholipids and the formation of an altered domain structure [27,35,36]. Bacterial membranes generally contain negatively charged phospholipids. Therefore, POGH, containing the positively charged guanidinium moieties and hydrophobic side chains, is likely to attach to the bacterial surface structure, make further efforts to insert into the phospholipids bilayer, and permeate the outer membrane, which causes local membrane holes and the release of cellular inclusions; subsequently, cell death followed. It was reported that polyhexamethylene diamine biguanide is rapidly adsorbed onto the outer surface of the negatively charged cells. It causes outer membrane damage, facilitates acidic phospholipids to form “membrane domain” structures on the cell membrane, and increases cell membrane permeability [37]. Chlorhexidine is easily absorbed by *E. coli* [37]. It was also suggested that cell death and cell membrane damage are direct consequences of antibacterial agents and are not mediated by intracellular autolytic enzymes [37]. Based on our data on beta-lactamase activity detection, confocal fluorescence microscopy, and scanning and transmission electron microscopy, we proposed that POGH is absorbed by the extracellular coating, influences the cell membrane, significantly impairs outer and inner membrane organization, increases membrane permeability, and induces the formation of local membrane holes. With the release of intracellular soluble substances, cells are induced to enter apoptosis. 

However, it is yet unclear whether the membrane disruption is the only bactericidal mode of action for POGH. In 2016, one related study reported that, contrary to the accepted model of microbial membrane disruption by polyhexamethylene biguanide (PHMB), PHMB enters both bacterial and mammalian cells, condenses bacterial chromosomes, and is excluded from mammalian nuclei, suggesting DNA binding as an alternative antimicrobial mechanism by using a PHMB-fluorophore conjugate together with cell growth, microscopy, and physiological assays [38]. In addition, transmission electron micrographs images demonstrated antimicrobial Polymyxin B, a typical membrane-targeted antibiotic fighting against MDR-PA and *Acinetobacter baumannii*, and induced a strong condensation or darkening of intracellular material and a more electron-dense peptidoglycan layer of *Acinetobacter baumannii* [39]. Similarly, in our case, dense, condensed particles and large aggregates were observed in the protoplast (Figure 6f–h) when cells were exposed to 30 μg/mL POGH, which indicated POGH could impair intracellular DNA and proteins. 

Indeed, POGH had an oral median lethal dose (LD50) ranging between 500 mg of polymer/kg of body weight and 800 mg/kg in this study. Based on the chemical hazard classification of the Environmental Protection Agency (Washington, DC, USA), POGH were in toxicity Category III with a LD50 range of 500 to 5000 mg/kg (oral administration to rats), slightly toxic. 

## 5. Conclusions

The mechanism of action of POGH against MDR-PA that involved the damage of the outer and inner membranes was dose-dependent. All doses of POGH within a range of 3–23 μg/mL increased the outer membrane permeability and subsequently facilitated the release of macromolecule enzyme proteins. A 30 μg/mL dose of POGH led to the separation of the inner and outer membrane, an increase in the membrane gap, and even the collapse of the outer membrane and the formation of local membrane holes. But it is unclear whether the membrane disruption is the only bactericidal mode of action for POGH. This study provided the microscopic examination of the disruption of the outer and inner membranes and intracellular structural changes in the representative-resistant bacteria-MDR-PA induced by the guanidine-based, antimicrobial polymer. The present microscopic information explained the strong outer-membrane permeation ability of guanidine-based antimicrobial polymers, which could be considered for the molecular design of novel guanidine-based polymers for combating antibiotics resistant to Gram-negative pathogens. 

## Abbreviations

The following abbreviations are used in this manuscript:

MDR-PA	multidrug resistant *Pseudomonas aeruginosa*
MRSA	methicillin-resistant *Staphylococcus aureus*
POGH	polyoctamethylene monoguanidine hydrochloride

## References

[1] Takahashi H., Caputo G.A., Vemparala S., Kuroda K. (2017). Synthetic random copolymers as a molecular platform to mimic host-defense antimicrobial peptides. Bioconjug. Chem..

[2] Kuroda K., Caputo G.A. (2013). Antimicrobial polymers as synthetic mimics of host-defense peptides. Wiley Interdiscip. Rev..

[3] Palermo E.F., Kuroda K. (2010). Structural determinants of antimicrobial activity in polymers which mimic host defense peptides. Appl. Microbiol. Biotechnol..

[4] Muñoz-Bonilla A., Fernández-García M. (2015). The roadmap of antimicrobial polymeric materials in macromolecular nanotechnology. Eur. Polym. J..

[5] Hurdle J.G., O’Neill A.J., Chopra I., Lee R.E. (2011). Targeting bacterial membrane function: An underexploited mechanism for treating persistent infections. Nat. Rev. Microbiol..

[6] Gorityala B.K., Guchhait G., Goswami S., Fernando D.M., Kumar A., Zhanel G.G., Schweizer F. (2016). Hybrid antibiotic overcomes resistance in *P. aeruginosa* by enhancing outer membrane penetration and reducing efflux. J. Med. Chem..

[7] Ouberai M., El Garch F., Bussiere A., Riou M., Alsteens D., Lins L., Baussanne I., Dufrene Y.F., Brasseur R., Decout J.L. (2011). The *Pseudomonas aeruginosa* membranes: A target for a new amphiphilic aminoglycoside derivative?. Biochim. Biophys. Acta Biomembr..

[8] Mingeot-Leclercq M.P., Decout J.L. (2016). Bacterial lipid membranes as promising targets to fight antimicrobial resistance, molecular foundations and illustration through the renewal of aminoglycoside antibiotics and emergence of amphiphilic aminoglycosides. MedChemComm.

[9] Pollard J.E., Snarr J., Chaudhary V., Jennings J.D., Shaw H., Christiansen B., Wright J., Jia W.Y., Bishop R.E., Savage P.B. (2012). In vitro evaluation of the potential for resistance development to ceragenin CSA-13. J. Antimicrob. Chemother..

[10] Shireen T., Singh M., Das T., Mukhopadhyay K. (2013). Differential adaptive responses of *Staphylococcus aureus* to in vitro selection with different antimicrobial peptides. Antimicrob. Agents Chemother..

[11] Álvarez-Paino M., Muñoz-Bonilla A., Fernández-García M. (2017). Antimicrobial polymers in the nano-world. Nanomaterials.

[12] Muñoz-Bonilla A., Fernández-García M. (2012). Polymeric materials with antimicrobial activity. Prog. Polym. Sci..

[13] Villanueva M.E., Gonzalez J.A., Rodriguez-Castellon E., Teves S., Copello G.J. (2016). Antimicrobial surface functionalization of PVC by a guanidine based antimicrobial polymer. Mater. Sci. Eng. C.

[14] Gilbert P., Moore L.E. (2005). Cationic antiseptics: Diversity of action under a common epithet. J. Appl. Microbiol..

[15] Wei D.F., Wang H., Ziaee Z., Chibante F., Zheg A.N., Xiao H.N. (2016). Non-leaching antimicrobial biodegradable PBAT films through a facile and novel approach. Mater. Sci. Eng. C.

[16] Ghamrawi S., Bouchara J.P., Tarasyuk O., Rogalsky S., Lyoshina L., Bulko O., Bardeau J.F. (2017). Promising silicones modified with cationic biocides for the development of antimicrobial medical devices. Mater. Sci. Eng. C.

[17] Choi H., Kim K.J., Lee D.G. (2017). Antifungal activity of the cationic antimicrobial polymer-polyhexamethylene guanidine hydrochloride and its mode of action. Fungal Biol..

[18] Donalisio M., Ranucci E., Cagno V., Civra A., Manfredi A., Cavalli R., Ferruti P., Lembo D. (2014). Agmatine-containing poly(amidoamine)s as a novel class of antiviral macromolecules: Structural properties and in vitro evaluation of infectivity inhibition. Antimicrob. Agents Chemother..

[19] Thakkar N., Pirrone V., Passic S., Zhu W., Kholodovych V., Welsh W., Rando R.F., Labib M.E., Wigdahl B., Krebs F.C. (2009). Specific interactions between the viral coreceptor CXCR4 and the biguanide-based compound NB325 mediate inhibition of human immunodeficiency virus type 1 infection. Antimicrob. Agents Chemother..

[20] Zhou Z.X., Wei D.F., Lu Y.H. (2015). Polyhexamethylene guanidine hydrochloride shows bactericidal advantages over chlorhexidine digluconate against ESKAPE bacteria. Biotechnol. Appl. Biochem..

[21] Bera S., Zhanel G.G., Schweizer F. (2010). Antibacterial activity of guanidinylated neomycin B- and kanamycin A-derived amphiphilic lipid conjugates. J. Antimicrob. Chemother..

[22] Grare M., Dibama H.M., Lafosse S., Ribon A., Mourer M., Regnouf-de-Vains J.B., Finance C., Duval R.E. (2010). Cationic compounds with activity against multidrug-resistant bacteria: Interest of a new compound compared with two older antiseptics, hexamidine and chlorhexidine. Clin. Microbiol. Infect..

[23] Zhou Z.X., Wei D.F., Guan Y., Zheng A.N., Zhong J.J. (2011). Extensive in vitro activity of guanidine hydrochloride polymer analogs against antibiotics-resistant clinically isolated strains. Mater. Sci. Eng. C.

[24] Oule M.K., Azinwi R., Bernier A.M., Kablan T., Maupertuis A.M., Mauler S. (2008). Polyhexamethylene guanidine hydrochloride-based disinfectant: A novel tool to fight meticillin-resistant *Staphylococcus aureus* and nosocomial infections. J. Med. Microbiol..

[25] Buxbaum A., Kratzer C., Graninger W., Georgopoulos A. (2006). Antimicrobial and toxicological profile of the new biocide Akacid plus^®^. J. Antimicrob. Chemother..

[26] Broxton P., Woodcock P.M., Heatley F., Gilbert P. (1984). Interaction of some polyhexamethylene biguanides and membrane phospholipids in *Escherichia coli*. J. Appl. Microbiol..

[27] Chawner J.A., Gilbert P. (1989). Interaction of the bisbiguanides chlorhexidine and alexidine with phospholipid vesicles: Evidence for separate modes of action. J. Appl. Microbiol..

[28] Li X.Z., Zhang L., Srikumar R., Poole K. (1998). β-Lactamase inhibitors are substrates for the multidrug efflux pumps of *Pseudomonas aeruginosa*. Antimicrob. Agents Chemother..

[29] Li X.Z., Poole K. (2000). Interplay between the MexA-MexB-OprM multidrug efflux system and the outer membrane barrier in the multiple antibiotic resistance of *Pseudomonas aeruginosa*. J. Antimicrob. Chemother..

[30] Lamers R.P., Nguyen U.T., Nguyen Y., Buensuceso R.N.C., Burrows L.L. (2015). Loss of membrane-bound lytic transglycosylases increases outer membrane permeability and beta-lactam sensitivity in *Pseudomonas aeruginosa*. MicrobiologyOpen.

[31] Mangoni M.L., Papo N., Barra D., Simmaco M., Bozzi A., Di Giulio A. (2004). Effects of the antimicrobial peptide temporin L on cell morphology, membrane and viability of *Escherichia coli*. Biochem. J..

[32] Epand R.F., Mowery B.P., Lee S.E., Stahl S.S., Lehrer R.I., Gellman S.H., Epand R.M. (2008). Dual mechanism of bacterial lethality for a cationic sequence-random copolymer that mimics host-defense antimicrobial peptides. J. Mol. Biol..

[33] Smith P.T., Huang M.L., Kirshenbaum K. (2015). Osmoprotective polymer additives attenuate the membrane pore-forming activity of antimicrobial peptoids. Biopolymers.

[34] Schneider V.A.F., Coorens M., Ordonez S.R., Tjeerdsma-van Bokhoven J.L.M., Posthuma G., van Dijk A., Haagsman H.P., Veldhuizen E.J.A. (2016). Imaging the antimicrobial mechanism(s) of cathelicidin-2. Sci. Rep..

[35] Zorko M., Jerala R. (2008). Alexidine and chlorhexidine bind to lipopolysaccharide and lipoteichoic acid and prevent cell activation by antibiotics. J. Antimicrob. Chemother..

[36] Chawner J.A., Gilbert P. (1989). Adsorption of alexidine and chlorhexidine to *Escherichia coli* and membrane components. Int. J. Pharm..

[37] McDonnell G., Russell A.D. (1999). Antiseptics and disinfectants: Activity, action, and resistance. Clin. Microbiol. Rev..

[38] Chindera K., Mahato M., Sharma A.K., Horsley H., Kloc-Muniak K., Kamaruzzaman N.F. (2016). The antimicrobial polymer PHMB enters cells and selectively condenses bacterial chromosomes. Sci. Rep..

[39] Girardello R., Visconde M., Cayo R., de Figueiredo R., Mori M.A.D., Lincopan N. (2017). Diversity of polymyxin resistance mechanisms among *Acinetobacter baumannii* clinical isolates. Diagn. Microbiol. Infect. Dis..

